# Editorial: Pharmacometrics—tools to assure optimal medicine use in low- and middle-income countries

**DOI:** 10.3389/fphar.2022.1034807

**Published:** 2022-10-06

**Authors:** Ahmed A. Abulfathi, Linda A. Chaba, Elin M. Svensson, Goonaseelan C. Pillai, Bernd Rosenkranz

**Affiliations:** ^1^ Center for Pharmacometrics and Systems Pharmacology, Department of Pharmaceutics, University of Florida, Orlando, FL, United States; ^2^ Department of Clinical Pharmacology and Therapeutics, College of Medical Sciences, University of Maiduguri, Maiduguri, Nigeria; ^3^ Division of Clinical Pharmacology, Department of Medicine, Faculty of Medicine and Health Sciences, Stellenbosch University, Cape Town, South Africa; ^4^ Strathmore Institute of Mathematical Sciences, Strathmore University, Nairobi, Kenya; ^5^ Department of Pharmaceutical Biosciences, Uppsala University, Uppsala, Sweden; ^6^ Department of Pharmacy, Radboud Institute for Health Sciences, Radboud University Medical Center, Nijmegen, Netherlands; ^7^ Division of Clinical Pharmacology, Department of Medicine, Faculty of Health Sciences, University of Cape Town, Cape Town, South Africa; ^8^ Fundisa African Academy of Medicines Development Cape Town, Cape Town, South Africa

**Keywords:** pharmacometrics, low- and lower-middle-income countries, pharmacokinetic/ pharmacodynamic (PK/PD), optimized dosage, communicable and noncommunicable diseases

Pharmacometrics is the science of using advanced mathematical and statistical methods based on biology, pharmacology, physiology, and knowledge of the pathophysiology of disease to quantify the relationships between the dose of an administered drug, its time course in the body, and its clinical efficacy and safety in individual patients and whole populations. This methodology optimizes therapy and has become an integral part of modern drug discovery and development and model-informed precision dosing at the patient’s bedside.

This Research Topic of Frontiers in Pharmacology aimed to address the application of pharmacometrics methodologies in medicines development, individual patient management, and medicines regulation in low- and middle-income countries (LMICs) and promote the discipline of pharmacometrics in LMICs, particularly in Africa. Speakers at the 2022 World Conference on Pharmacometrics (WCoP 2022), held in Cape Town, South Africa, were encouraged to submit manuscripts. The central theme of the three accepted and published manuscripts is infectious diseases, two of which focused on tuberculosis (TB). The fourth published manuscript focused on the placebo effect in pain studies.

The first manuscript (Abulfathi et al.) described first-time data on the population pharmacokinetics of meropenem in patients with TB. Meropenem is being repurposed for the treatment of TB. The ultimate purpose of the investigation by Abulfathi et al. was to optimize the clinical use of meropenem in patients with TB. The optimization requires the understanding of meropenem’s pharmacokinetic/pharmacodynamic index linked to its anti-TB effect. A first step to achieving this goal was to develop a two-compartment model with first-order elimination from the central compartment that adequately the pharmacokinetics of meropenem. Allometric scaling using total body weight on disposition parameters and creatinine clearance improved the model’s predictive performance. It is reassuring that meropenem coadministration with rifampicin, a potent inducer of drug-metabolizing enzymes and transporters did not alter meropenem pharmacokinetics. The model could be used in an integrated pharmacokinetic-pharmacodynamic analysis linking meropenem exposures to early bactericidal activity against TB, and simulations investigated the probability of target attainment with various dosing regimens.

The second manuscript (Munir et al.) showed that a one-compartment model with first-order elimination could describe the pharmacokinetics of vancomycin in post-operative Pakistani patients. The inclusion of creatinine clearance explained between-subject variability in vancomycin exposures and allowed dose-individualization based on the degree of renal impairment.

The third manuscript (Van der Laan et al.) described lamivudine and abacavir pharmacokinetics in human immunodeficiency virus-infected children with and without multidrug-resistant TB (MDR-TB) treatment. In this South African study, lamivudine and abacavir exposures were not significantly altered by co-administration with commonly used drugs for MDR-TB including terizidone, ethambutol, ethionamide, high-dose isoniazid, pyrazinamide, amikacin, and fluoroquinolones. However, due to the small sample size, no conclusions could be drawn for several individual medications. Newer anti-TB drugs such as bedaquiline and delamanid were not included because they were not yet available to treat MDR-TB in children at the time of the study. The authors recommended prospective studies to evaluate drug-drug interactions between antiretroviral drugs and the increasing number and regimens of anti-TB drug combinations, including moxifloxacin, levofloxacin, clofazimine, linezolid, bedaquiline, and delamanid.

The fourth manuscript (Sun et al.) pooled data across two randomized, placebo-controlled trials in patients with neck pain due to cervical radiculopathy, and investigated the potential covariates that might predict a placebo effect. The authors developed a multivariate logistic regression model and found that the covariates age and sex had no significant effect. They were able to identify pain duration of fewer than 2 weeks as a risk factor for a placebo effect.

Several authors intending to submit manuscripts for peer review withdrew because even the discounted article processing costs (APC) were unaffordable. This indicates a need for mechanisms that enable the publication in open-source, high-impact factor journals of the rising high-quality research from LMICs. The Journal of Antimicrobial Chemotherapy-Antimicrobial Resistance and several BMC journals as part of Springer Nature, etc., waive completely the APC for qualifying LMICs. These waivers are encouraging and should herald the widespread adoption of this policy for a part of the global community carrying the highest burden of both infectious and non-communicable diseases. Advocacy at all levels is required to reduce inequality in science and global health.

